# Collinear search impairment is luminance contrast invariant

**DOI:** 10.1038/s41598-021-90909-w

**Published:** 2021-06-01

**Authors:** Chia-huei Tseng, Hiu Mei Chow, Jiayu Liang, Satoshi Shioiri, Chien-Chung Chen

**Affiliations:** 1 Research Institute of Electrical Communication, Tohoku University, Sendai, Japan; 2 Department of Ophthalmology and Visual Sciences, University of British Columbia, Vancouver, Canada; 3 Department of Psychology, University of Hong Kong, Pok Fu Lam, Hong Kong; 4 Department of Psychology, National Taiwan University, Taipei, Taiwan

**Keywords:** Sensory processing, Human behaviour

## Abstract

Collinear search impairment (CSI) is a phenomenon where a task-irrelevant collinear structure impairs a target search in a visual display. It has been suggested that CSI is monocular, occurs without the participants’ access to consciousness and is possibly processed at an early visual site (e.g. V1). This effect has frequently been compared with a well-documented opposite effect called attentional capture (AC), in which salient and task-irrelevant basic features (e.g. color, orientation) enhance target detection. However, whether this phenomenon can be attributed to non-attentional factors such as collinear facilitation (CF) has not yet been formally tested. Here we used one well-established property of CF, i.e. that target contrast modulates its effect direction (facilitation vs suppression), to examine whether CSI shared similar signature profiles along different contrast levels. In other words, we tested whether CSI previously observed at the supra-threshold level was reduced or reversed at near-threshold contrast levels. Our results showed that, regardless of the luminance contrast levels, participants spent a longer time searching for targets displayed on the salient singleton collinear structure than those displayed off the structure. Contrast invariance suggests that it is unlikely that CSI is exclusively sub-served by an early vision mechanism (e.g. CF).

## Introduction

Visual search is a daily task at which we have to excel. Our visual system relies on a quick computation to direct our mental focus efficiently towards the region that requires most processing, and the idea of “salience map” has been proven to be a conceptually and quantitatively convenient approach for capturing the way that our attention is deployed across visual space^1^. Although the detailed computation parameters and rules may differ, most salience models include an initial stage where separate analyses of basic feature information (e.g. color, orientation, and motion) take place before a later stage where these streams of information are integrated^2,3^. Recently, a mid-layer of computation based on grouping principles has received growing attention.

Grouping principles are laws that describe how we segregate visual scenes into figure and ground^4^, and these principles, although readily perceived, have not been adopted as formal components of a visual attention model. However, several lines of research have indicated that a perceptually grouped region is advantageous in terms of target discrimination^5–7^. For example, targets displayed within a closure were detected faster than when they were outside the closure^8–10^, suggesting that grouping modulates attentional priority. Gabor patterns located within a continuous curvature were easier to detect than those located away from a curvature^6,11^.

**Figure 1:**
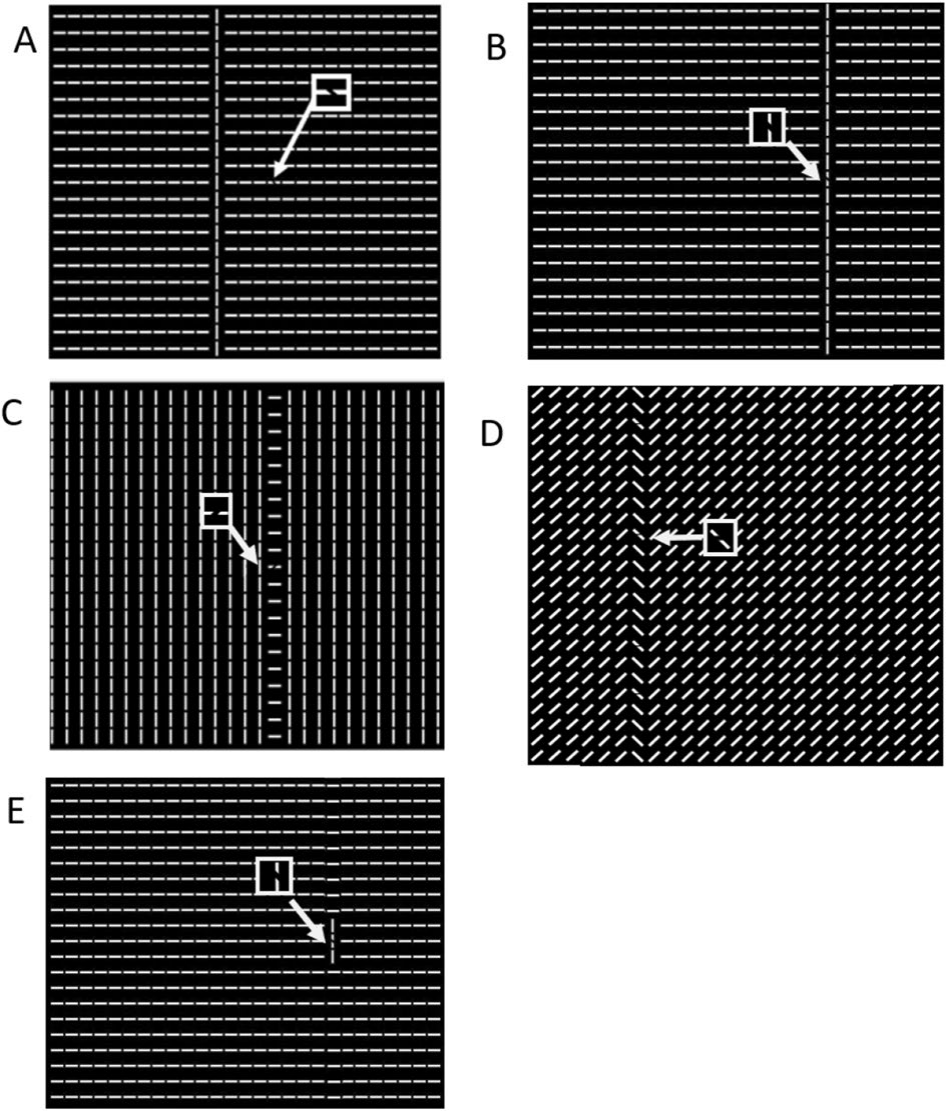
Visual search displays with (**A**) a non-overlapping target and (**B**–**E**) an overlapping target on a (**B**) full-length collinear structure, (**C**) ladder-like/non-collinear structure, (**D**) slant structure, and (**E**) short collinear structure.

Counterintuitively, a recent series of studies reported a disadvantage of target orientation discrimination when the target spatially overlapped a collinear structure^12,13^. When observers had to make a two-alternative forced choice as to whether a target tilt was counterclockwise or anti-counterclockwise, their performance was impaired when targets appeared on a salient (yet task-irrelevant) structure consisting of collinear bars (Fig. 1B) than off it (Fig. 1A). Note that the target is a break along a texture line (not the texture line itself). It is important to also point out that even though the target in Fig. 1A does not overlap with the salient (vertical) collinear distractor column, it is embedded within a horizontal collinear structure. However, the target and its locating line segment in Fig. 1A are less visually salient than those in Fig. 1B. This is because all elements adjacent to the target in Fig. 1A are in parallel following the same horizontal collinearity, while in Fig. 1B all the neighboring elements are orthogonal. Past studies have suggested that visual search impairment effect only occurred when the task-irrelevant distractor is a salient singleton column as in Fig. 1B^12^. Moreover, this search deterioration seems to be limited to collinear grouped structures and was absent when the grouped structure was organized in a ladder shape (Fig. 1C), as slant stripes (Fig. 1D), or when the collinear structure was short (Fig. 1E).

Several possible high-level contributions to this effect have been ruled out by Tseng and Jingling^13^. A series of experiments were conducted to demonstrate that factors such as strategic suppression over an area with a low occurrence rate by the learning of statistical regularity^14–16^, or interference by the matched features between distractor with the main task (i.e. top-down control settings)^17,18^ were unlikely to be the cause of this search performance reduction. While this collinear impairment of visual search is immune to high-level cognitive modulation, whether or not it is a consequence of an early perceptual limitation has not been directly tested. A good candidate to account for the collinear search impairment is the collinear flanker effect. The early experiments examining collinear facilitation used a set of collinear bars^19^, similar to the collinear structure used by Chow et al^20,21^. The collinear flanker effect refers to the change in visual response to an oriented target in the presence of nearby oriented stimuli (flankers) that are collinear with the target. For instance, the detection threshold of a line segment^19^ or a Gabor pattern^22^ decreases with the presence of collinear line segments or Gabor flankers, respectively. Electrophysiology^23–28^ and fMRI^29^ evidence also shows that the neural response to a target at V1 can be modulated by the presence of collinear flankers.

The strengths of the collinear facilitation effect and collinear search impairment both depend on the configurations. For example, the collinear flanker effect is most prominent when the flankers are collinear with the target without orientation^22,30^, spatial frequency^22,31^, or phase^32^ offset. Such configuration dependency was also found for collinear search impairment. The orientation alignment of a structure is also of critical importance. When the task-irrelevant distractor is formed into a ladder shape (i.e. Fig. 1C), search impairment disappears^12^. When the task-irrelevant collinear structure is short or only a singleton (i.e. a single bar only), the observers’ performance is unaffected, or can be enhanced in some cases of a singleton^13,33^.

Furthermore, neither phenomenon requires the participants’ conscious awareness of the full collinear structure. Hayashi and Murakami^34^ made oriented flankers invisible through interocular suppression so that the flankers were masked from the other eye by random patterns. Significant collinear facilitation was still reported. The collinear flanker effect is often considered to result from lateral interactions between visual neurons^23–29^. An attention account of the collinear flanker effects suggests that the flankers provide a cue to the target location, reducing spatial uncertainty and improving target detection performance^35,36^. However, this theory can be rejected based on the fact that collinear facilitation still exists even when an observer is provided with complete knowledge about the subsequent target location^37^. Thus, attention and conscious awareness of the collinear structure also have little influence on the collinear facilitation effect. Instead, these reports support the view that these functions may be served by preconscious and automatic processing. In terms of collinear search impairment, Chow and Tseng^38^ used dynamic noise patterns to mask out part of the collinear structure so that the visible parts were shorter than the critical length for provoking search impairment. Nevertheless, significant search impairment has been robustly reported, which indicates that the subjective awareness of a complete collinear structure is not required. Similarly, Chow, Jingling, and Tseng^20^ used a singleton column defined by the eye-of-origin to provoke similar search impairment. As the participants were unable to identify the origin of the eye where the stimuli were displayed (i.e. they had no access to the ocular origin), it implies that the access of consciousness is not critical as regards this observed phenomenon.

In the current study, we use the contrast effect to examine whether collinear search impairment is an extended effect of collinear facilitation. The collinear flanker facilitation effect is known to be contrast dependent. Polat et al.^25^ (also see^24,39^) showed that the collinear flankers increase the response of V1 cortical neurons to a low contrast target and suppress their response to a high contrast target. At the behavioral level, Chen and Tyler^30,40,41^ showed that the collinear flankers, while facilitating target contrast discrimination at low contrast, increase the contrast discrimination threshold at high contrast. Hence, the collinear effect actually has two components: collinear facilitation at low contrast and collinear suppression at high contrast.

In all reported cases of collinear search impairment (e.g.^20,21,42^), the stimuli were high contrast (i.e. all well above the participants’ detection and discrimination thresholds). Thus, it is possible that collinear search impairment is caused by collinear suppression. Although the target (an oriented bar placed on a line segment) itself is not strictly speaking part of a collinear segment, it is located on a line segment belonging to a collinear structure. Participants’ success in identifying the line segment is likely to be advantageous in terms of target orientation discrimination (the main task).

Another established property of collinear facilitation is that it is influenced by the orientation of flankers of two sides of a target. Solomon and Morgan (2000) reported that extra iso-orientation flankers on two sides of a target can cancel the collinear facilitation effect from the flankers at the two ends of the target (similar to our non-overlapping condition in Fig. 1A)^43^. It is important to note that both the target and the collinear flankers in Solomon and Morgan’s study were at near-threshold contrast levels. It is unclear whether similar effects apply to high-contrast stimuli as there was no such study conducted to the best of our knowledge.

If we reduce the search display contrast levels to near-threshold, we expect two changes. First, there is weaker lateral inhibition^25^ from iso-orientation flankers at the two ends of the target (i.e. collinear suppression) or even a facilitatory effect (i.e. collinear facilitation). This applies to both non-overlapping (Fig. 1A) and overlapping conditions (Fig. 1B) because the target in Fig. 1A is part of a horizontal collinear structure while the target in Fig. 1B is part of a vertical collinear structure. Secondly, contrast reduction leads to weaker inhibition from iso-orientation at the two sides of the target as in Solomon and Morgan^43^ in non-overlapping condition (Fig. 1A), but not in overlapping condition (Fig. 1B) because the flankers on two sides of the target are of orthogonal orientation and do not modulate the target detection^44^. The combined effect would reduce, remove, or reverse the collinear search impairment, defined as the performance (accuracy or RT) differences between overlapping and non-overlapping conditions. If this is the case, it would suggest that collinear search impairment is an extension of collinear flanker effects.

## Methods

### Participants

Thirteen students from National Taiwan University (NTU) and twenty-four students from the University of Hong Kong (HKU) were recruited. They were compensated with additional course credits or 30 HKD (for HKU)/120 NTD (for NTU). They had normal or corrected-to-normal vision and had not participated in any previous experiments. All the participants provided written informed consent to participate in the experiment. This study was approved by the Ethics Committee of the University of Hong Kong and National Taiwan University. The experiment was carried out in accordance with the Code of Ethics of the World Medical Association (Declaration of Helsinki).

### Stimuli and apparatus

The experiment was conducted in a dimly lit room. The computer program for experimental control was written in Matlab with Psychtoolbox^45,46^.

**Figure 2:**
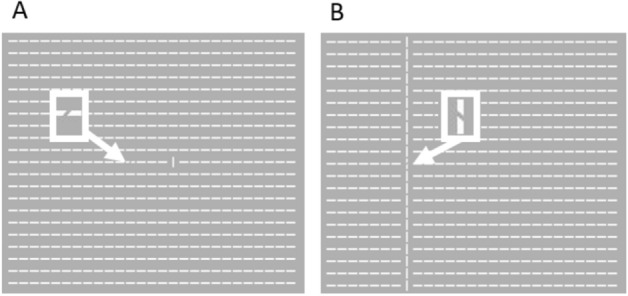
Visual search displays. (**A**) The distractor is short (size = 1 bar), and the non-overlapping target tilts to the right. (**B**) The distractor is full length (size = 21 bars) and the overlapping target tilts to the left.

The search display consisted of 567 white element bars arranged in 21 rows × 27 columns against a gray background (Fig. 2). Each bar had a visual angle of 0.40° × 0.07°. The target was a gap (45° tilted right or left) in one textured element bar (Fig. 2). All the bars were horizontal except for one salient column comprising 1 (Fig. 2A) or 21 vertical bars (Fig. 2B), randomly selected in each trial.

At NTU, the stimuli were presented on an Eizo 24-inch LCD monitor controlled by a Macintosh computer via a Radeon 7200 graphics board, which provided a 10-bit digital-to-analog converter depth. The input voltage-output intensity function of the LCD monitor was measured with a PhotoResearch PR 655 radiometer. The display had a mean luminance of 34.4 cd/m^2^, and the refresh rate of the monitor was 60 Hz. The viewing distance was set such that each pixel subtended a visual angle of 1 min of arc. At HKU, the monitor used was a 17-in. LCD (Philips Brilliance 190 s, 40.9 × 25.4 cm) driven by an NVIDIA GeForce 8600 GT with a grayscale resolution of 8 bits per pixel. The resolution of the monitor was 1600 × 1200 pixels with a 60-Hz refresh rate, and the mean luminance was 26.9 cd/m^2^ (measured with the same radiometer). At a viewing distance of 70 cm, the size of each pixel was 1 min of arc per screen pixel.

The bars had four different luminance intensity levels that were 1.7%, 7.5%, 15.5%, and 19.5% higher than the background luminance. These levels were determined from a pilot study to yield near-threshold responses because this would create space for us to observe the facilitation and inhibition of interest. As we expect to see both search impairment and facilitation effects (based on different collinear configurations), it is critical to ensure that we avoid the ceiling effect (i.e. the contrast level exceeds the threshold by too much) and floor effect (i.e. sub-threshold contrast levels). Each intensity level contained an equal number of trials (i.e. 144 trials), randomized for each trial. The numbers of short (size = 1 bar) and full-length (size = 21 bars) distractors were the same for each intensity (i.e. 72 trials).

### Design and procedures

The target was always presented at the center (i.e. 11th) row and randomly at one of the six columns on either side of the central column (− 6th, − 4th, − 2nd, 2nd, 4th, 6th column in relation to the central column). The distractor column was also randomly placed in one of the same six possible locations. An overlapping target was defined as one where the target gap was located on the distractor column (Fig. 1B), and a non-overlapping target referred to the targets shown on other columns (e.g. Fig. 1A). We independently varied the target and the distractor location so that only one sixth of the trials contained overlapping targets (i.e., chance level, because there were six possible locations). Thus, the distractor column had no power to predict the target location and therefore was irrelevant to the task.

Each trial began with a fixation circle centered on the display for 800 ms, followed by the target display, which stayed on the screen until the participant responded or the response time limit of 5 s had passed. This criterion was set based on previous studies which showed that average response time of this type of task was under 2 s. Trials requiring more than 5 s to respond were excluded from analysis. Subjects were asked to indicate whether the gap tilted left or right from vertical by pressing one of the two keys. Practice trials were given to the subjects until they could respond correctly for both overlapping and non-overlapping conditions. In practice trials, the bar intensity was 100%, so the task was much easier than that in the real experiment. Participants were instructed to respond as quickly as possible while maintaining accuracy. During the experiments, participants were required to take a break after every 96 trials. Each participant completed 576 trials which took approximately 30 min.

## Results

**Figure 3:**
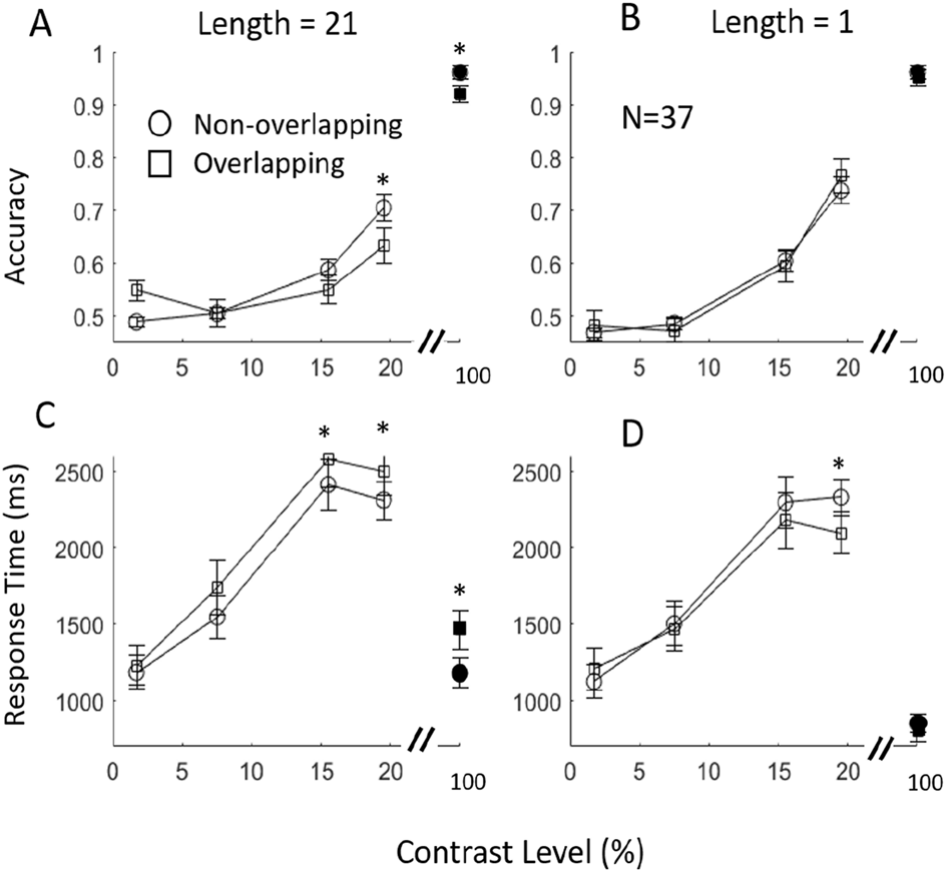
Accuracy (**A**,**B**) and response time (**C**,**D**) results (open symbols) with standard error. Participants’ responses for a search display with a long distractor column (**A**,**C**) indicated a search impairment at contrast levels near detection threshold (75% accuracy performance). This result is similar to those reported for supra-threshold levels (100% condition in current graph, solid symbols) by Tseng and Jingling^13^. In contrast, there was no such impairment under conditions with a short distractor length (**B**,**D**). *p < 0.05.

Figure 3 shows how accuracy and response time (RT) changed with luminance contrast for all participants. The results for the high contrast (100%) version of the corresponding conditions reported by Tseng and Jingling^13^ are also plotted here for comparison. Under all these conditions, RT first increased with luminance contrast up to 15.5%, and then showed signs of a decrease as the luminance contrast further increased (Fig. 3C,D). However, at the highest contrast that we measured (19.5%), the RTs remained higher than with the maximum (near 100%) high-contrast conditions described by Tseng and Jingling^13^. When a vertical collinear structure was included in the stimuli (line length = 21), the RT for a target embedded in the collinear structure (overlapping) was significantly longer than that for a target embedded in the background (non-overlapping), t(36) = 1.74, p = 0.04, at 19.5% luminance contrast. This result is similar to that obtained with the high contrast conditions tested in a previous study^13^. When there is no collinear structure (line length = 1), the RT result showed the opposite trend. That is, the RT for the target in a background element (non-overlapping) was slower (t(36) = 1.90, p = 0.03, at 19.5% contrast). Again, this is consistent with high contrast results^13^.

The accuracy (Fig. 3A,B) increased with luminance contrast, from the chance level at the two lowest contrasts to about 70% when the target overlapped the salient collinear distractor structure and to about 80% when target did not overlap the salient collinear structure. These results are much lower than those in the previous study with high contrast conditions, which easily reached 95–100%^13^. This confirmed that the luminance contrast of our stimuli was indeed near our target discrimination threshold (75%). We analyzed the accuracy to determine whether the collinear RT impairment was in exchange for high accuracy (i.e. speed-accuracy tradeoff) but found no evidence of a speed-accuracy tradeoff. The accuracy for targets embedded in the collinear structure was significantly lower than that in a background element (t(36) = 2.23, p = 0.015). Furthermore, the accuracy result also helped us understand why the RT increased with luminance contrast. At a very low contrast (1.7%, 7.5%), where the participants’ performance is near the chance level (50%), the participants’ responses were fast, possibly because they were unable to see the stimuli and thus press a response key quickly without processing anything. As the luminance contrast increased, the participants started to perceive the stimuli and made an effort to process them, and this was reflected in an increased RT. Thus, it is not surprising that the maximum RT in our experiment was higher than that reported for high contrast stimuli by Tseng and Jingling^13^.

Although averaged results based on contrast levels suggested that our selected contrast levels were appropriate choices for probing near-threshold performance, a closer look at the data revealed noticeable individual differences in both the overall performance and the contrast level yielding near-threshold performance. As it was critical to include only trials near the threshold (accuracy ~ 75%), we employed a performance-based analysis in two steps. First, we excluded data from participants whose overall performance for all the trials (i.e. both overlapping and non-overlapping conditions) fell outside of the threshold range [66.67%, 83.31%]. This range was computed as the 95% confidence interval with standard derived from a binomial distribution. Data from participants whose performance was above the upper range (i.e. the saturation range of the psychometric function) and below the lower range (i.e. near the chance level) were not informative for near-threshold performance analysis. As a result, of the participants we tested, only 25 (15 from NTU, and 10 from HKU) met the criteria and were included in the subsequent additional analysis. Second, for each participant, we selected the contrast level where his or her near-threshold performance was observed. The selected 25 participants were tested at all 4 contrast levels (1.7%, 7.5%, 15.5%, and 19.5%), but the near-threshold performance for each individual subject might actually fall at different contrast levels.

**Figure 4:**
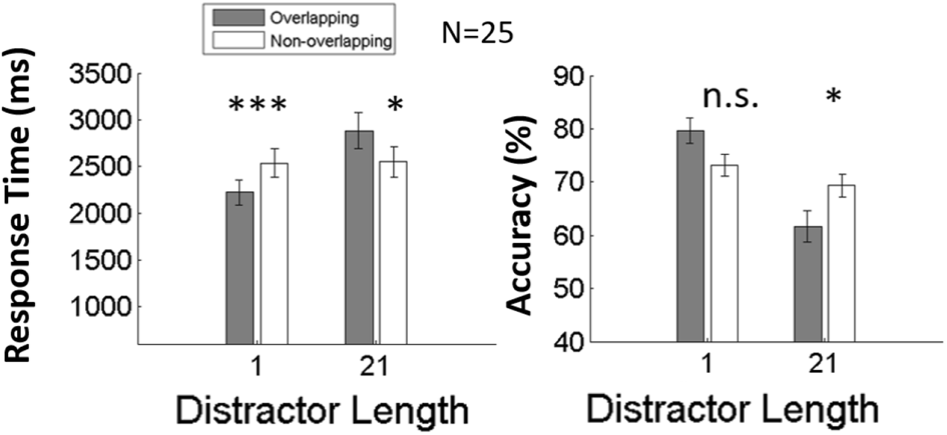
Experimental results of near-threshold performance from 25 participants who met a stricter performance criterion to have their performance within the range [66.67%, 83.31%]. Error bars denote standard errors. ***p < 0.001 and *p < 0.05.

Figure 4 plots the RT results based on near-threshold performance analysis. The following analysis was based on the selected 25 participants’ near-threshold performance only (i.e. only 1 contrast level or 25% of trials). The RT from accurate trials was submitted to two (distractor size: 1 and 21 bars) by two (target type: overlapping and non-overlapping) repeated measures ANOVA. The RT was longer when the search display contained a full-length distractor (2.72 s) than when it contained a short distractor (2.38 s) (F(1, 24) = 13.6, p < 0.001).

There was a significant interaction between distractor size and target type (F(1, 24) = 13.5, p < 0.001). Tukey’s post-hoc analysis showed that at full length (21 bars), search RTs for targets overlapping the distractor were significantly impaired (2.88 s vs. 2.55 s, t(24) = 3.63, p < 0.05), while at a short length (1 bar), overlapping targets received a search advantage (2.22 s vs. 2.54 s, t(24) = 3.45, p < 0.05) (t(24) = 7.23, p < 0.001). There was no significant difference with respect to target type (F(1, 24) = 0.007, p = 0.933). The accuracy analysis revealed a consistent pattern as with the RT analysis; accuracies were higher for short distractors (76.4%) than for long distractors (65.5%) (F(1, 24) = 25.9, p < 0.001) and there was a significant interaction (F(1, 24) = 12.1, p < 0.01). Tukey’s post-hoc analysis revealed impaired accuracies on overlapping targets when a full-length distractor (size = 21) was present (61.7% vs. 69.3%, t(24) = 3.38, p < 0.05). We found no significant difference for target type (F(1, 24) = 0.043, p = 0.838). The results did not suggest a speed-accuracy trade-off.

As a control, we analyzed trials at the near chance level (defined as 50% accuracy, occurred at the lowest contrast level if there were more than one contrast levels satisfied this criterion) for the same 25 participants. We found no significant differences for target types (F(1, 24) = 0.069, p = 0.795), distractor length (F(1, 24) = 0.487, p = 0.492), or interaction between the two (F(1, 24) = 0.227, p = 0.638).

## Discussion

We investigated the mechanism underlying collinear visual search impairment by testing the hypothesis that a contrast dependent low-level visual limitation contributed to this impairment. We adjusted the stimuli search display from supra-threshold to near- threshold, which should remove the collinear suppression effect. The results showed that participants still required longer time to search for visual targets on the collinear structure than off the collinear structure (i.e. collinear search impairment) near the threshold performance level. Thus, the mechanisms underlying search impairment should be either independent of luminance contrast or saturated even at the near-threshold level.

This result is inconsistent with the idea that the collinear search impairment is caused by collinear suppression. For instance, the response of a V1 neuron to a high contrast Gabor pattern target can be suppressed by the presence of high contrast flankers collinear with the target^25,39^. A psychophysical result also shows that the flankers can enhance the visibility of a low contrast target but suppress it when the target is superimposed on a high contrast pattern^40^. On the other hand, extra flankers at the sides, as is the case when the target is in the background in our experiment, can cancel out the collinear suppression^43^. Thus, visual search impairment, which was discovered with high contrast stimuli^12,13^, may well be the result of such collinear suppression. However, the collinear flanker effect is contrast dependent. Collinear flankers suppress the neural responses to a high contrast target but facilitate the response for low contrast targets^25,39^. Such a cross-over effect was also reported at the behavior level, where the presence of collinear flankers can reduce the contrast increment threshold at low contrast but increase it at high contrast^30,40,41,47^. However, our result shows that collinear search impairment also occurs at low near-threshold contrast levels. Reducing the contrast does not turn impairment into improvement. Thus, collinear search impairment does not share mechanisms with the collinear flanker effect.

We next consider whether the end-stopping response property of visual cortical neurons^48,49^ may underlie the collinear search impairment. For cells with end stopping properties, their response first increases with the length of a small target to a critical value then decreases as the length of the target *increases* further^48^. Thus, a target embedded in a linear structure may produce a lower response in the visual system than the target itself due to the end-stopping from the extended linear structure. However, neurophysiological studies have shown that the end-stopping response is also contrast dependent; the same cells that show an end-stopping property for high contrast stimuli also show no response reduction for a length increment of low contrast stimuli^50^. Thus, if end-stopping underlies collinear search impairment, we should observe the reverse of the effect at low contrast. Yet, our data did not show such a property.

An alternative candidate is collinear filling-in. A recent optical imaging study on monkeys showed that collinear flankers produce neural activity in the visual cortex at the target location when the target is not present, and this was taken as electrophysiological evidence for a filling-in effect^51^, possibly through lateral interactions even if the feedforward input does not directly activate the corresponding target location^52–54^. Behaviorally, the target location can also be “filled-in” from the flankers, for example, when the target-to-flanker distance is short i.e. 0–2 wavelength units (the distance between two light stripes of the Gabor pattern)^22,55^. Our search target in this study was not a Gabor pattern but the parameters may be well within the parameter ranges for the production of filling-in. Sakaguchi^56^ discovered a contrast-dependence effect when a 1.2° horizontal Gabor pattern was placed in a surrounding region filled by a 12-degree diagonal sinusoidal grating (i.e. 45° orientation difference). The perceptual filling-in time increased when the stimuli contrast levels increased, which could lead to a prediction that perceptual filling-in is weaker at high contrast, thus contrast reduction would make the target detection and search harder (e.g. bigger search impairment). However, a closer look at Sakaguchi’s study^56^ revealed a noticeable interaction between target and surround contrasts. Participants’ perceptual filling-in time increased only when target contrast was above the surround contrast (fixed at 10%, 25%, 50%), but unaffected when it was equal or below the surround contrast levels. Our search display has always contained elements of equal contrast levels. Therefore, a direct generalization of Sakaguchi’s contrast finding to our current study might be problematic. In addition, perceptual filling-in was more prominent in peripheral vision than central vision^57^. Our attentional search task was a free-viewing task, and targets fell on a very small bar (0.40° × 0.07°). It was most plausible that the participants performed the orientation discrimination task in or near the fovea region, where perceptual filling-in was hard to observe within a short duration (participants’ responses were made within 2 s). For these reasons, perceptual filling-in is an unlikely candidate for such an effect.

We believe that collinear search impairment may be best understood from a model that includes two contrast-independent stages. In the first stage, participants need to locate a target embedded among other distractors and grouping textures (target localization; the ‘where’). Under such circumstances, participants might experience interference between local/global regions. An eye-tracking study result^58^ suggests that targets on a collinear structure (overlapping condition, Fig. 1B) attract fewer initial saccades than those off a collinear structure (non-overlapping condition, Fig. 1A). As a short-latency saccade is usually taken to be the reflection of bottom-up saliency, this behavioral result implies an early disadvantage in target localization. In the second stage, participants determine the target orientation and make reports (target orientation discrimination; the ‘what’). The search impairment can originate from this stage as well. For example, attention priority setting studies^17,18^ report the spill-over attentional effect to features which are not goal-related. When an observer needed to discriminate between a red target T and L, a salient item (e.g. a target location cue) not directly to the goal (e.g. letter discrimination) could draw attention only when its color matched the target color (e.g. red), not when it was in a mismatched color (e.g. white). In our case, target orientation discrimination might be interfered with contextual collinearity along the feature domain. Our past studies have suggested that the search impairment is not limited to orientation discrimination task only but present in a variety of task requirements including target detection, target localization, color discrimination, shape discrimination, and luminance discrimination. Thus, the attention priority setting might not be the sole player. Another possible factor is attentional focus. Our target was a small item and local attention was required to report its detail feature. The salient background collinear structure might expand partcipants’ attentional focus, hindering their ability to deply local attention. The effects from the two stages may not be exclusive. After the first stage, collinear interference might be further amplified when the target properties must be determined. The detailed mechanisms of the contextual influence remain unclear, but we have ruled out a few possibilities because of its contrast-invariant property revealed in the current study.
